# Comparison of the Wild-Type Obligate Methylotrophic Bacterium *Methylophilus quaylei* and its Isogenic Streptomycin-Resistant Mutant via Metal Nanoparticle Generation

**DOI:** 10.1007/s12011-019-01740-4

**Published:** 2019-05-09

**Authors:** Vladimir V. Sorokin, Anna B. Pshenichnikova, Sergei V. Kalenov, Nikolay A. Suyasov, Dmitry A. Skladnev

**Affiliations:** 1 Winogradsky Institute of Microbiology, Research Center of Biotechnology of the Russian Academy of Sciences, 33, Bld. 2 Leninsky Ave., Moscow, Russia; 2 Department of Biotechnology and Industrial Pharmacy, MIREA – Russian Technological University, 86 Vernadsky Avenue, Moscow, Russia; 3 Department of Biotechnology, Faculty of Biotechnology and Industrial Ecology, D.I. Mendeleyev University of Chemical Technology of Russia, 9 Miusskaya Square, Moscow, Russia

**Keywords:** Silver nanoparticles, Palladium nanoparticles, Methylotrophic bacterium, Biogenic nanoparticles

## Abstract

Metal nanoparticles synthesized by green methods with the use of microorganisms are currently one of the most closely studied types of nanomaterials. It has accurately been shown that the characteristics of metal nanoparticles generated in the presence of different bacteria vary. For the two isogenic strains of obligate methylotrophic bacteria of the wild type (*M. quaylei* MT^T^) and its streptomycin-resistant mutant (*M. quaylei* SM^R^), the pleiotropic character of streptomycin resistance mutation in the SM^R^ cells has been revealed. It has been shown that both cultures can generate silver nanoparticles. There is a dramatic difference in the formation of palladium nanoparticles, which are formed only in the presence of cells of the streptomycin-resistant mutant *M. quaylei* SM^R^. This study shows that closely related isogenic strains of obligate methylotrophic bacteria can be distinguished by the spectra of biogenic nanoparticles of two noble metals. While palladium nanoparticles are only generated by the cells of the streptomycin-resistant mutant *M. quaylei* SM^R^, biogenic silver nanoparticles can be generated from both cultures. Thus, the assessment of the ability of microorganisms to form biogenic nanoparticles of different metals allows the revelation of subtle metabolic differences of even close cultures.

## Introduction

Methylotrophic bacteria are well known as objects of various biochemical and biotechnological studies [1–4] but not as producers of biogenic metal nanoparticles. At the same time, gram-negative aerobic methylotrophic bacteria have prospects for use in nanobiotechnology for the biogenic synthesis of metal nanoparticles. Obligate methylotrophic microorganisms are non-pathogenic because they can not receive the sole carbon and energy source for growth in a mammalian organism and it’s important for production purposes. Perhaps that is why methylotrophic microorganisms have been considered as industrial, biotechnological objects in the past few decades [5–8]. The high growth rate, the low price of growth media with methanol, and the plasticity of the regulation of growth parameters make it possible to consider methylotrophs as promising universal producers.

Methylotrophic cells can use soluble periplasmic methanol dehydrogenase, which is an enzyme containing the prosthetic pyrroloquinoline quinone (PQQ) group. The methanol dehydrogenase of methylotrophs catalyzes the first reaction in an unusual periplasmic electron transport chain responsible for the oxidation of methanol to formaldehyde, utilizing the mechanism of the direct transfer of a hydride ion from the methyl group of methanol to PQQ [1]. Alternative electron acceptors in such a chain can be cations that are present in the medium, which are the source material for the formation of nanoparticles of the corresponding metals [9–12].

In cases of excessive content of toxic cations in the medium, the formation of nanoparticles from reduced atoms as a less toxic product is observed as a natural protective reaction of microbial cells [13–15]. It is believed that the ability to form nanoparticles of metals from sterile salt solutions artificially introduced into cell suspensions can be considered to be a complex characteristic of the natural ability of the microorganisms for self-preservation [16, 17]. The possibility of using the process of the formation of biogenic silver nanoparticles for a comparative integral evaluation of the biological properties of microorganisms was first shown by the example of different microbial communities in the horizons of the Antarctic subglacial Lake Untersee [18]. In all the samples studied, the formation of Ag**°**NPs from an artificially introduced sterile silver salt solution occurred only in the presence of microbial cells. In the control analogs, specifically in the corresponding water samples that were released from the bacterial cells, the generation of nanoparticles was not observed because of the absence of cation reductants in the oligotrophic medium of the samples. The authors named this method of studying the cells of microorganisms the DBNG (detection of biogenic nanoparticles generation) [18, 19].

It has been proposed to use the ability of microorganisms to generate metal nanoparticles from reduced cations as an indicator of the presence of metabolically active cells in test samples [19]. Since it is known that the external components of microbial cells play the primary role in the recovery of cations [20–23], this study compared the ability of closely related analogs of cultures of microorganisms that differ in the characteristics of the surface structures of their cells to generate biogenic nanoparticles of noble metals.

To compare closely related microorganisms using the DBNG method, a widely used approach in genetics was chosen. The approach consists of a comparative analysis of an isogenic pair of cultures. One of the cultures differs from the other in that it contains a single additional mutation leading to some change in the properties of the original culture. In this study, the DBNG method used the cells of an isogenic pair of the obligate methylotrophic bacterium *M. quaylei*, the wild type, and an isogenic streptomycin-resistant mutant, to compare the ability to form biogenic nanoparticles. Previously, the ability to compare an isogenic pair of cultures to synthesize metal nanoparticles had never been the subject of nanobiotechnological research.

It should be noted that, similar to the oligotrophic microorganisms of Lake Untersee, the cultures of the obligate methylotroph *M. quaylei* require simple synthetic media for growth that do not contain high-molecular-weight organic compounds that can participate in the chemical reduction of silver cations during the formation of nanoparticles. The absence of abiogenic reductants makes it possible to more accurately assess the details of the interaction of the salts with the cells of closely related methylotrophic bacteria.

## Materials and Methods

### The Cultures of the Microorganisms

The obligate methylotrophic bacteria deposited in the all-Russian collection of microorganisms (VKM), specifically, *M. quaylei* (VKM B-2338) of the wild type [24] and its isogenic streptomycin-resistant SM^R^ mutant, were used.

The genetic characterization of the cultures of an isogenic pair of obligate methylotrophic *M. quaylei* strains was conducted using a standard sequence analysis of their 16S rRNA genes [25].

### Cultivation of *M. quaylei*

The methylotrophic cultures were grown on MQ medium, including a mineral source of nitrogen and phosphates to stabilize the pH level, with the following composition (g L^−1^): K_2_HPO_4_ 1.5, KH_2_PO_4_ 0.7, NaNO_3_ 1, MgSO_4_×7H_2_O 0.2, and CaCl_2_ 0.02. Two milliliters of trace element solution was added separately and contained (mg 100 mL^−1^) FeSO_4_×7H_2_O 100, ZnSO_4_×7H_2_O 5, MgCl_2_×4H_2_O 1.5, CoCl_2_×6H_2_O 10, CuCl_2_×5H_2_O 5, NiCl_2_×6H_2_O 1, Na_2_MoO_4_ 1.5, and EDTA 250. The components were sterilized in solution at 121 °C for 25 min. The phosphates and trace element solutions were sterilized separately. All the solutions cooled quickly and were mixed with the trace element solution and methanol (0.5%, *v*/*v*) as the sole carbon and energy source under sterile conditions.

Cultivation was carried out at 28 °C and 180 rpm using a Unimax 2010 orbital platform shaker (Heidolph, Germany) within 3 days. The volume of the media was 50 mL, with cultivation in 250 mL conical flasks. Experiments of the NP generation and sensitivity of bacteria to silver and palladium salts were conducted using cultures in the exponential phase at 9 × 10^8^ CFU/ml.

### ζ-Potential Measurement

Measurement of cell surface ζ-potential was performed with a Delsa Nano Submicron Particle Size Analyzer (Beckman Coulter Inc., USA) that uses electrophoretic light scattering for the ζ-potential determination with the cells which are washed and placed in a buffer of a predetermined ionic strength and pH value.

### Materials

As the source of ionic silver, an aqueous solution of Ag(NH_3_)_2_NO_3_ synthesized from AgNO_3_ and ammonia according to a modified Tollens protocol was used to form the Ag**°**NPs [19, 26]. The sterile Ag(NH_3_)_2_NO_3_ solution was added directly to the samples to achieve a final concentration of 0.1 mM.

The source of the palladium ions for the formation of Pd**°**NPs was an aqueous solution of Na_2_PdCl_4_ synthesized from PdCl_2_ in a reaction with excess NaCl [27–29]. Sterile Na_2_PdCl_4_ solution was added directly to the samples to achieve a final concentration of 0.5 mM. The sterility of the aqueous solutions of Ag(NH_3_)_2_NO_3_ and Na_2_PdCl_4_ was confirmed by the absence of growth on the complex agar medium LB.

### Sensitivity of Bacteria to Silver and Palladium Salts

The sensitivity of fresh cell suspensions of the methylotrophic bacteria *M. quaylei* to silver and palladium salts was conducted in 1 mL liquid medium at room temperature (24–26 °С) without mixing. After 20-min incubation, the cell suspension was plated on solid agar medium. The sensitivity level was determined by the decrease in the number of CFU of the treated samples compared to the CFU of the control suspensions.

### NPs Formation

The generation of the biogenic AgNPs and PdNPs from the sterile salt solutions in all the experiments was conducted in 50-μL reaction mixtures with aliquots of cell suspension samples within 20 min, at pH 7–7.2 and temperature of 24–26 °C.

**Fig. 1:**
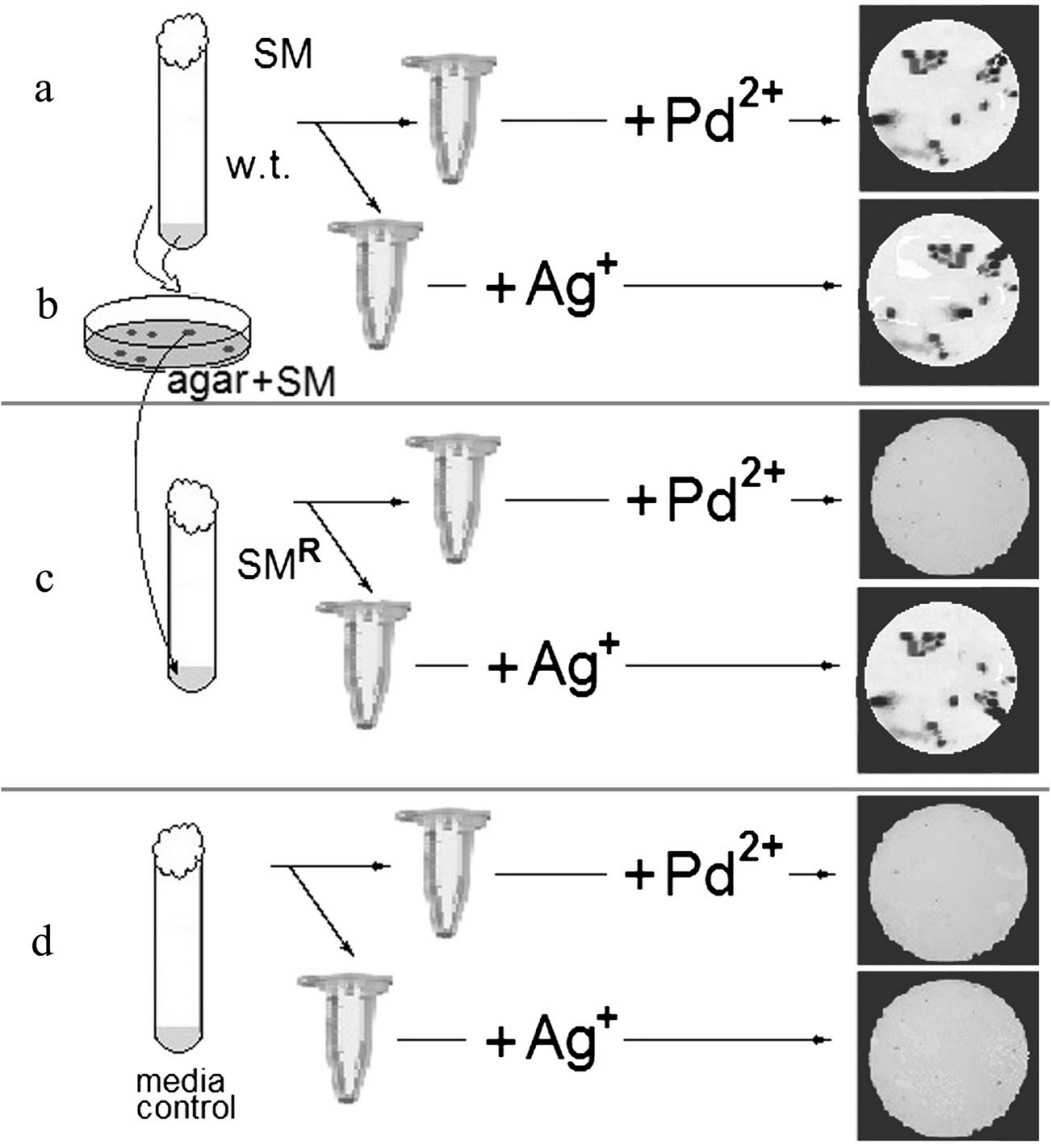
Experiments on the generation of silver and palladium NPs with different strains of *M. quaylei*. **a** To obtain nanoparticles, the salts of Ag^+^ or Pd^2+^ were added to suspensions of *M. quaylei* MT^T^ of wild type (w.t.). **b** To obtain a streptomycin-resistant mutant of *M. quaylei* SM^R^, the strain of *M. quaylei* MT^T^ was used. **c** To obtain nanoparticles, the salts of Ag or Pd were added to suspensions of *M. quaylei* SM^R^. **d** In the control variants, the salts of Ag^+^ or Pd^2+^ were added to the sterile growth medium. The pictures of NPs formed are shown to the right

Five microliters of the reaction mixture was transferred to the grids for transmission electron microscopy. After 5 min of sorption of the cells and NPs, the liquid was removed, and the grids were washed five times with double-distilled water and dried for 15 h. Aliquots of the samples from which the microorganisms were removed by centrifugation (10,000×*g*, 25 min) were used as the control [18, 19]. The absence of bacteria in the centrifuged control samples was confirmed by electron microscopy and by the absence of growth on MQ and LB agar media. The absence of NPs in the solutions and samples was confirmed by transmission electron microscopy. The scheme of the experiment is shown in Fig. 1.

### Transmission Electron Microscopy

The study of the morphology, linear sizes, and localization of nanoparticles was performed using transmission electron microscopy (TEM). Electron microscopy and X-ray microanalysis of the specimens were conducted using a JEM-1400 microscope (JEOL, Japan) equipped with an X-ray microanalyzer (Oxford Instruments, UK) at an accelerating voltage of 80 keV. The specimens were prepared using standard copper grids covered with Formvar and reinforced with carbon.

### X-ray Diffraction Analysis

The X-ray diffraction analysis (XRD) of NPs was fulfilled using XRD-6000 diffractometer (Shumadzu, Japan) under following conditions: Cu Kα_1_ irradiation in the range of 2θ angles from 30° to 80° with a step of 0.02° and exposure of 30 s at a point. The samples were previously dried and triturated in an agate mortar. Metallic silver and palladium were identified by the peaks in accordance with JCPDS 04-0783 for Ag^0^ and JCPDS 05-0681 for Pd^0^.

### Determination of NP Sizes

The size distribution of NPs and their characteristics were determined using TEM with the subsequent processing and analysis of images using LabVIEW IMAQ Vision (National Instruments, USA), and the analysis took into account no less than 1000 NPs.

## Results

### Streptomycin Resistance of Cells from Two Strains of *M. quaylei*

The streptomycin-resistant mutant *M. quaylei* SM^R^ was obtained earlier with protection from contamination during the mass cultivation of *M. quaylei* wild-type MT^T^ bacteria. For the parent strain of the wild-type *M. quaylei* MT^T^, the LD_50_ concentration of streptomycin is 0.005–0.01 mg mL^−1^, while the resistant mutant *M. quaylei* SM^R^ grows up to a concentration of 4 mg mL^−1^ in the presence of this antibiotic.

The pleiotropic character of the streptomycin resistance mutation of *M. quayle*i SM^R^ strain is manifested in the difference in properties of the surface biopolymers of cells. In the cells of the mutant *M. quaylei* SM^R^, there was a fivefold decrease in the secretion of exopolysaccharides, primarily down to 0.2 g L^−1^, while in the parent culture of *M. quaylei* MT^T^, this value was 1.05 g L^−1^. The values of the *ζ-*potential for the cultures of *M. quaylei* MT^T^ and *M. quaylei* SM^R^ are negative throughout the entire pH range, which indicates a negatively charged cell surface. In addition, the culture of *M. quaylei* SM^R^ is characterized by a low hydrophobicity of the cell surface (5.5%) compared to the increased hydrophobicity of the surface in the cells of the original strain (39%) (Table 1).

**Table 1:** Difference in the characteristics of nanoparticles and their synthesis in pair of isogenic strains

Characteristics of cultures	*M. quaylei* МТ^Т^		*M. quaylei* SM^R^	
EPS secretion, g L^−1^	1.05		0.2	
Cell surface hydrophobicity, %	39		5.5	
Characteristics of process	Average NPs size, nm	Cell survival, %	Average NPs size, nm	Cell survival, %
Ag^+^→Ag**°**NPs	45	32	70	0.4
Pd^2+^→ Pd**°**NPs	No Pd**°**NPs	5	70	59

The determination of the cell sensitivity of *M. quaylei* strains to silver and palladium salts was conducted in liquid synthetic growth medium with methanol, i.e., under conditions corresponding to those in which NP generation was performed with these metals. The survival of the bacteria in a 0.1 mM solution of Ag(NH_3_)_2_NO_3_ was 32% and 0.4% for the *M. quaylei* strains MT^T^ and SM^R^, respectively. When 0.5 mM Na_2_PdCl_4_ solution was added to the suspension of bacteria, the survival of the *M. quaylei* MT^T^ and SM^R^ strains was reduced to 5% and 59%, respectively (Table 1).

### Generation of Silver and Palladium NPs with the Obligate Methylotrophic Bacterium *M. quaylei*

Experiments on the synthesis of the NPs were conducted with actively growing cells, which were grown in a liquid synthetic growth medium with methanol and were taken directly from the flasks. The formation of nanoparticles was carried out by “green synthesis,” that is, directly in the culture medium with cells in the logarithmic growth phase. Under such conditions, both the cells themselves (their surface biopolymers) and the compounds secreted by the cells can play the role of cation reducing agents *in situ*.

**Fig. 2:**
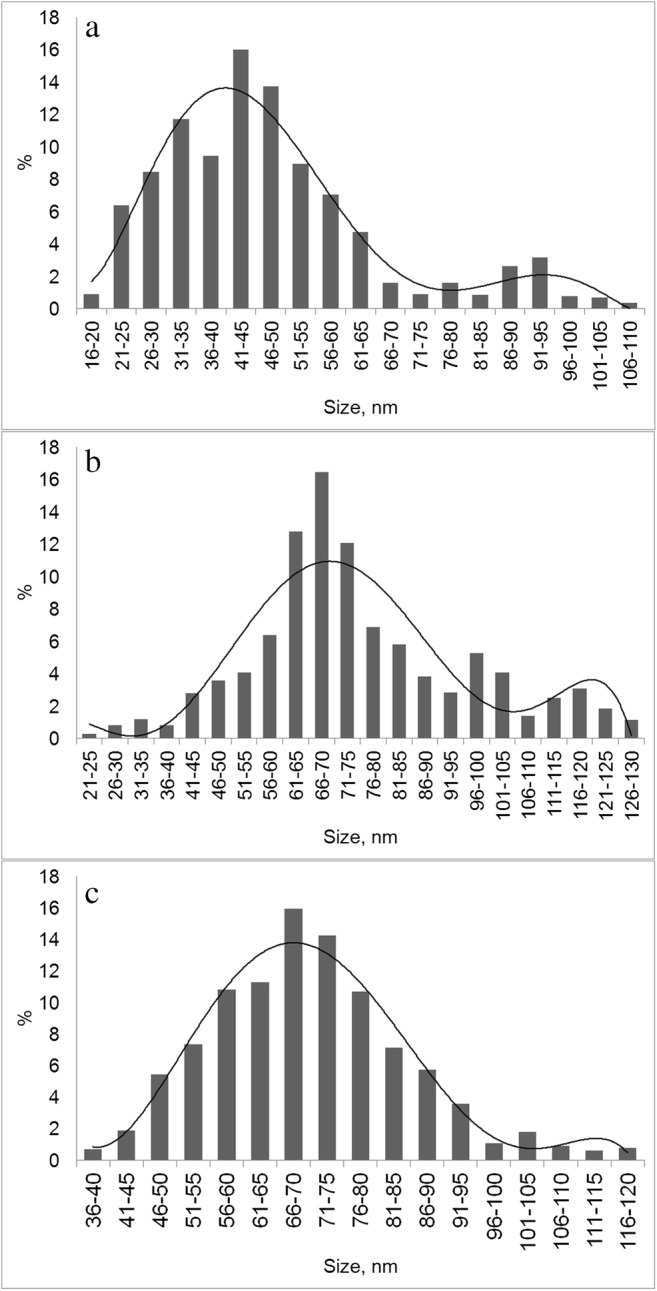
Size distribution of Ag**°**NPs obtained with *M. quaylei* MT^T^ (**a**) and *M. quaylei* SM^R^ (**b**) and size distribution of Pd**°**NPs obtained with *M. quaylei* SM^R^ (**c**)

**Fig. 3:**
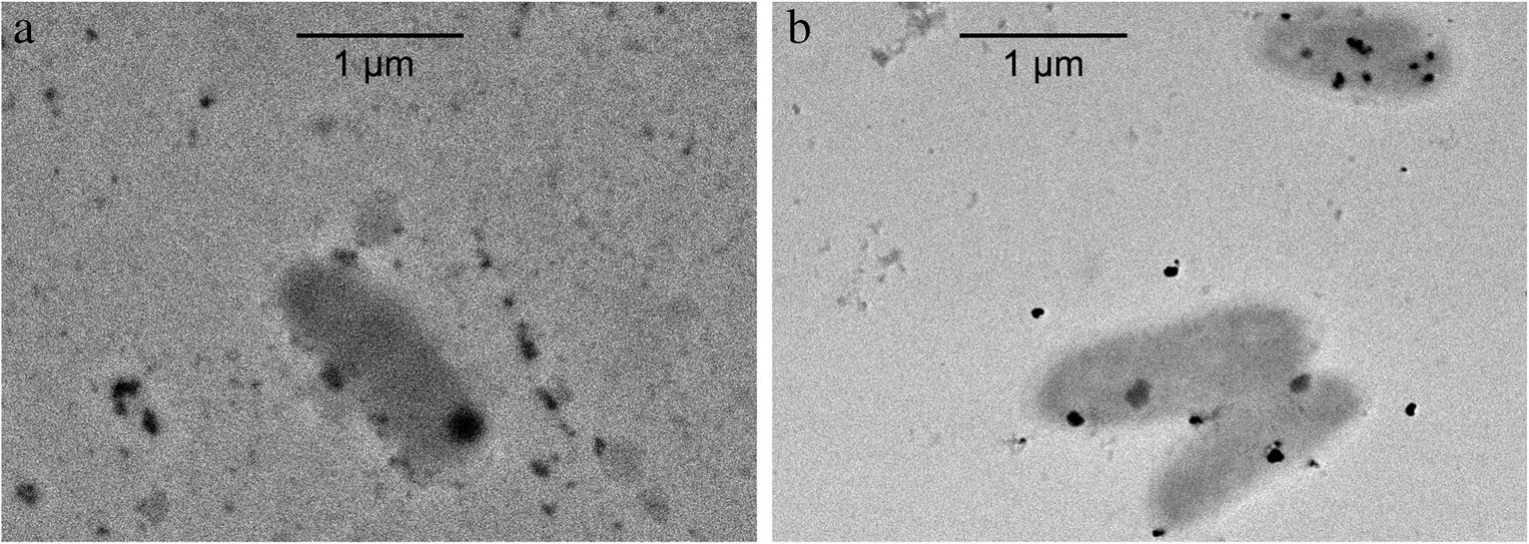
TEM of *M. quaylei* MT^T^ (**a**) and *M. quaylei* SM^R^ (**b**) cells with silver NPs

**Fig. 4:**
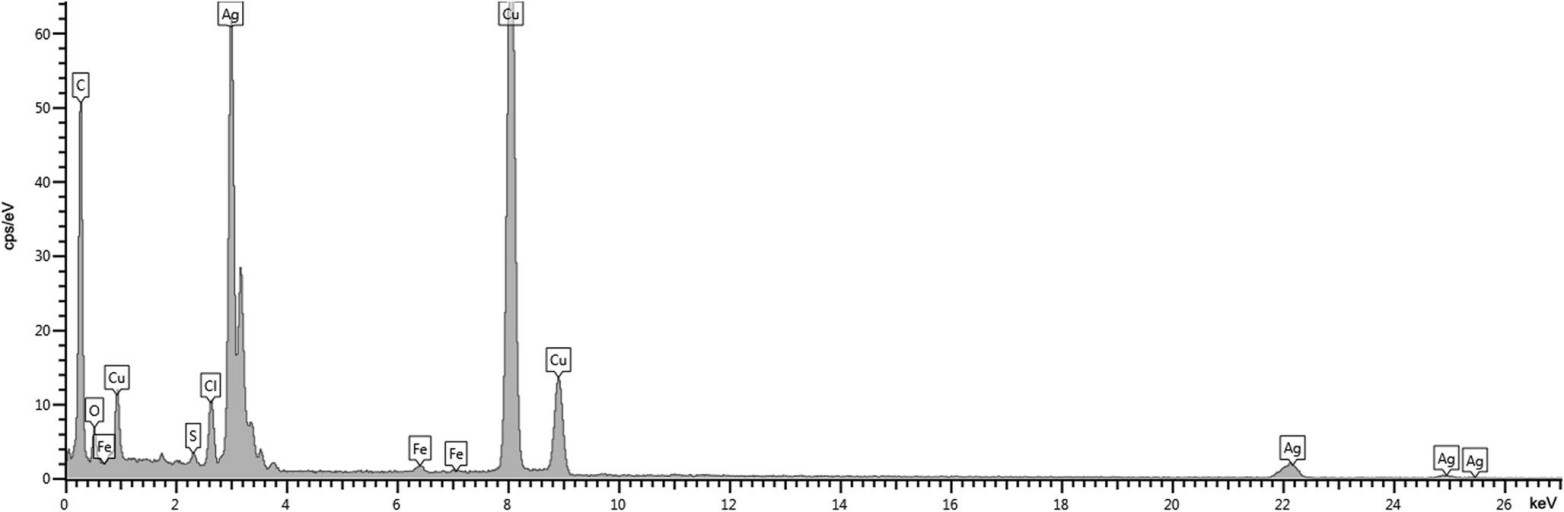
Typical EDS spectrum from regions of Fig. 3 with Ag**°**NPs

Both cultures of methylotrophic *M. quaylei* could form Ag**°**NPs when a sterile solution of Ag(NH_3_)_2_NO_3_ was added to the suspension. The Ag**°**NP sizes in the suspension of the wild-type *M. quaylei* MT^T^ culture cells were from 16 to 100 nm, with the primary fraction of 45 nm. At the same time, in the presence of cells of the streptomycin-resistant mutant *M. quaylei* SM^R^, Ag**°**NPs from 25 to 120 nm in size with the primary fraction of approximately 70 nm were formed (Figs. 2, 3, and 4).

**Fig. 5:**
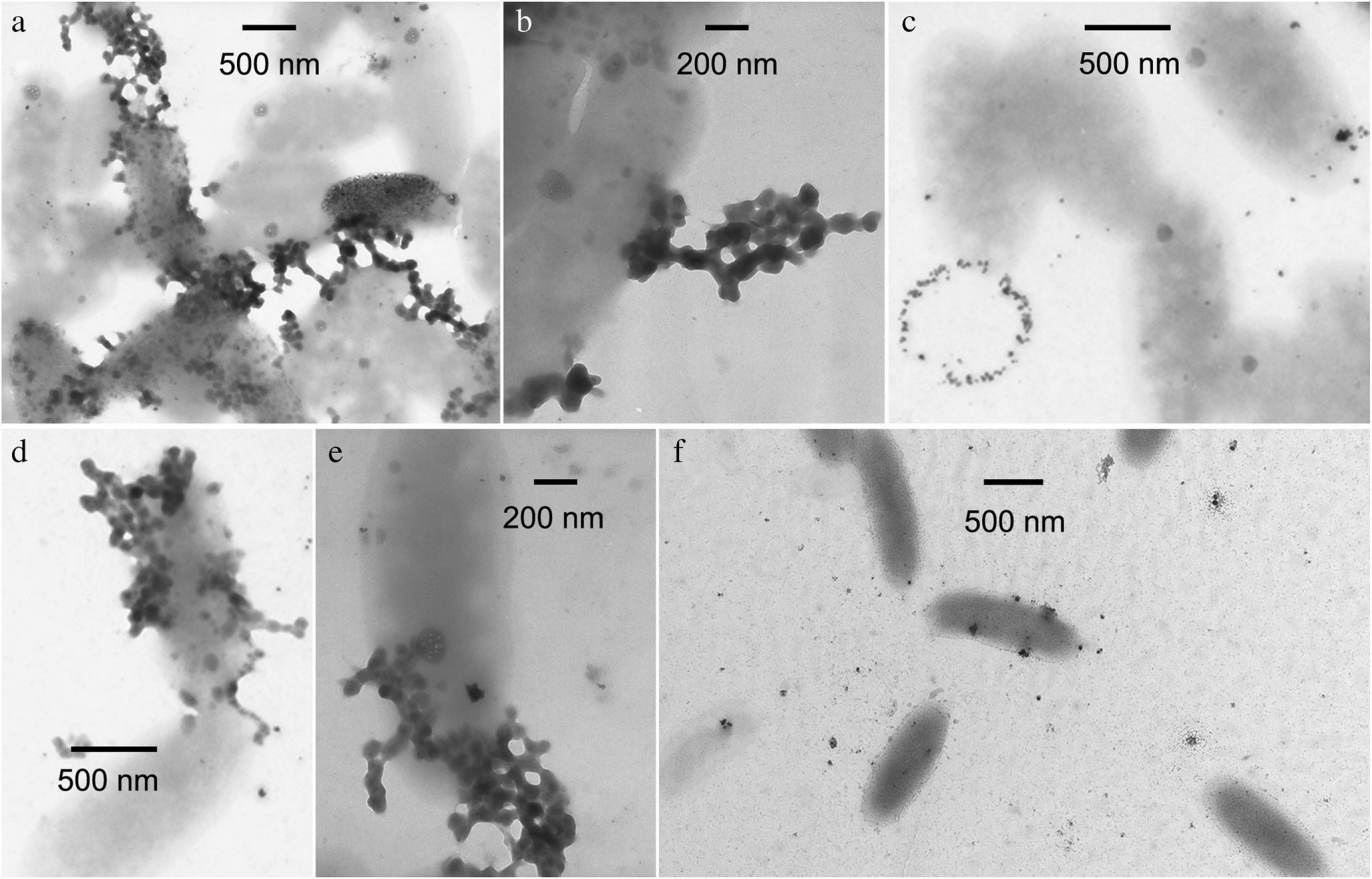
TEM of *M. quaylei* SM^R^ (**a**–**e**) cells with different palladium NPs clusters and TEM of *M. quaylei* MT^T^ (**f**) cells with small structures without reduced palladium

**Fig. 6:**
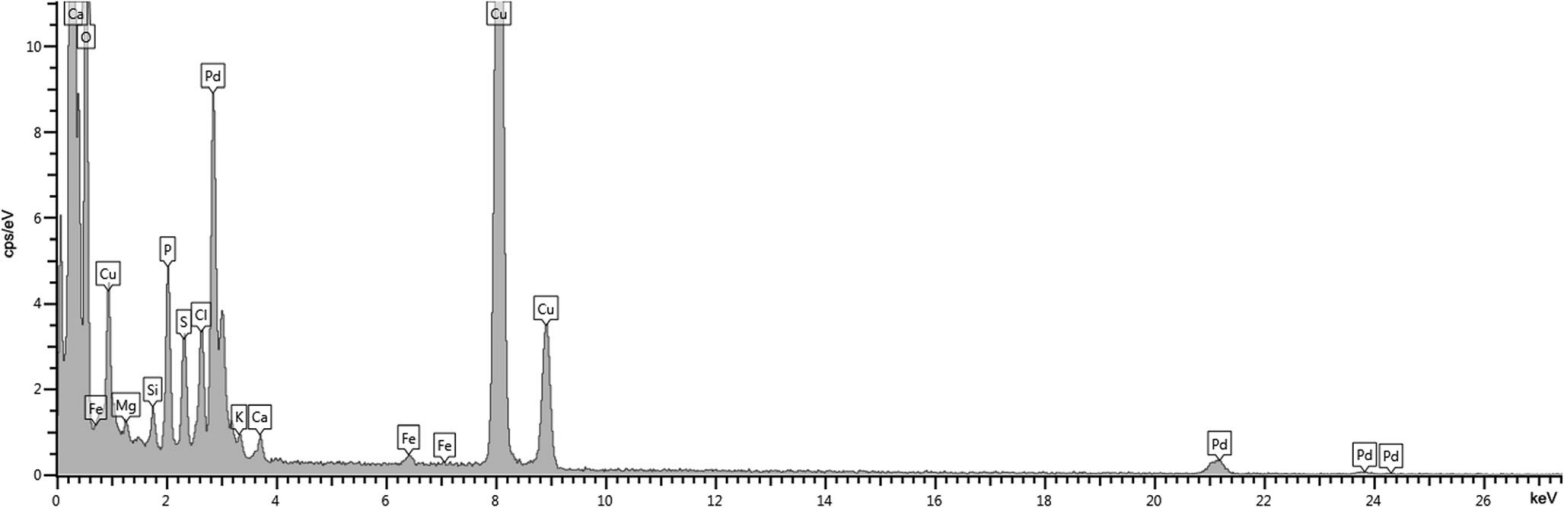
Typical EDS spectrum from regions of Fig. 5 with Pd**°**NPs

When a sterile solution of Na_2_PdCl_4_ was added to a suspension of *M. quaylei* methylotroph cells, Pd**°**NPs up to 70 nm in size were formed only in the presence of the streptomycin-resistant mutant *M. quaylei* SM^R^ (Figs. 2, 5, and 6). In the culture of the wild-type *M. quaylei* MT^T^, a significant number of optically opaque nanoscale objects were observed, which did not contain palladium according to X-ray microanalysis.

## Discussion

The study of the mechanisms of the antibiotic resistance of certain microorganisms remains a topic of medicine, molecular biology, and microbiology [16, 30–32]. The fact that the surface structures of the cells play a key role in the resistance to most antibiotics has been revealed for many types of microorganisms. In addition, studies devoted to the comparative study of cell resistance to the toxic effects of salts and the corresponding metal NPs on the pure cultures of various microorganisms were conducted [33–36]. It should be noted that one of the most informative techniques of the functional comparative studies of microbial cultures is the comparison of the properties of the isogenic pairs of microbial cultures. Isogenic pairs of cultures differ only by one feature, for example, modification by a mutation such as increased resistance to an antibiotic. However, this approach is generally not applied to the ability of isogenic cultures to form NPs [37, 38].

It was shown that the resistance to streptomycin in most isolates (up to 70–80%) with such a phenotype (SM^R^) is determined by mutations affecting the bacterial ribosomal protein S12 and 16S rRNA, encoded by *rps*L or *rrs* and 7-methyl-guanosine (m^7^G) methyltransferase *gid*B, respectively [30, 39, 40]. From the earliest studies, it was known that streptomycin-resistant derivatives are characterized by the pleiotropic nature of the manifestation of the mutations. In addition, streptomycin-resistant mutants often also showed additional unpredictable resistance to other antibiotics [41]. In this study, we compared for the first time the ability of a pair of isogenic cultures of the obligate methylotrophs *M. quaylei* (one of which is a streptomycin-resistant derivative of the other) to protect against the biocidal influence of noble metal salts in a growth medium by the formation of biogenic nanoparticles. We compared the effect of silver Ag(NH_3_)_2_NO_3_ and palladium Na_2_PdCl_4_ salts on the cells and observed the formation of biogenic nanoparticles of reduced silver and palladium. The pleiotropic nature of the streptomycin resistance mutation in *M. quaylei* SM^R^ was known previously [24]. The most noticeable differences were in the characters affecting the surface biostructures of the cells. These aspects include a fivefold decrease in the secretion of the exopolysaccharides (up to 0.2 g L^−1^) and an extremely low hydrophobicity of the cell surface (5.5%) compared to the hydrophobicity of the cells of the original *M. quaylei* MT^T^ strain, which is increased to 39% (Table 1). It was interesting to estimate the effect of such notable changes in the properties of the surface biopolymers of the *M. quaylei* cultures on the ability of the cells to reduce metal cations. For such comparisons of the reducing ability of microorganisms, noble metals were chosen, primarily silver and palladium, which differ in the valency of the cations [42–44]. The use of the pair of obligate methylotrophic *M. quaylei* strains in the study appeared to be highly rational, since a simple synthetic medium was used to cultivate the bacteria. This medium does not contain a variety of organic compounds that may have reducing abilities and further affect the process of generating nanoparticles.

In preliminary experiments with methylotrophs, it was shown that at salt concentrations of 0.1 mM Ag(NH_3_)_2_NO_3_ and 0.5 mM Na_2_PdCl_4_, a sufficiently pronounced biocidal effect is achieved while maintaining the cell viability level acceptable for producing biogenic nanoparticles (Table 1). Under these conditions, for the first time, significant differences in the features of the formation of reduced silver and palladium nanoparticles in the presence of a pair of isogenic cultures of obligate methylotrophs directly in synthetic growth media with methanol were observed. Thus, in the streptomycin-resistant mutant *M. quaylei* SM^R^, the intensive formation of palladium nanoparticles occurred. The average size of the Pd°NPs was 70 nm. Most of these nanoparticles were adsorbed on cells or occurred in the form of chains that connected the individual cells together (Fig. 5a). In contrast, in the bacterial suspension of the original *M. quaylei* MT^T^ strain, with a 20-min contact with a solution of the palladium salt, there was a complete absence of biogenic palladium nanoparticles. This result occurred with the background decrease in the cell survival to 5%. At the same time, a significant number of small structures were observed on the TEM image, in which there was no detectable number of atoms of the reduced palladium (Fig. 5f). Taking into account the pleiotropic nature of the streptomycin-resistant mutation in *M. quaylei* SM^R^ bacteria, it can be assumed that the actively synthesized exopolysaccharides of *M. quaylei* MT^T^ very quickly come into contact with palladium ions Pd and therefore selectively block the formation of Pd°NPs. This statement is supported by data on the high reactivity of Pd^2+^ cations towards biopolymers [45–47]. Thus, from the two isogenic cultures of methylotrophic bacteria, the ability to generate Pd°NPs manifests only in the streptomycin-resistant *M. quaylei* SM^R^.

**Fig. 7:**
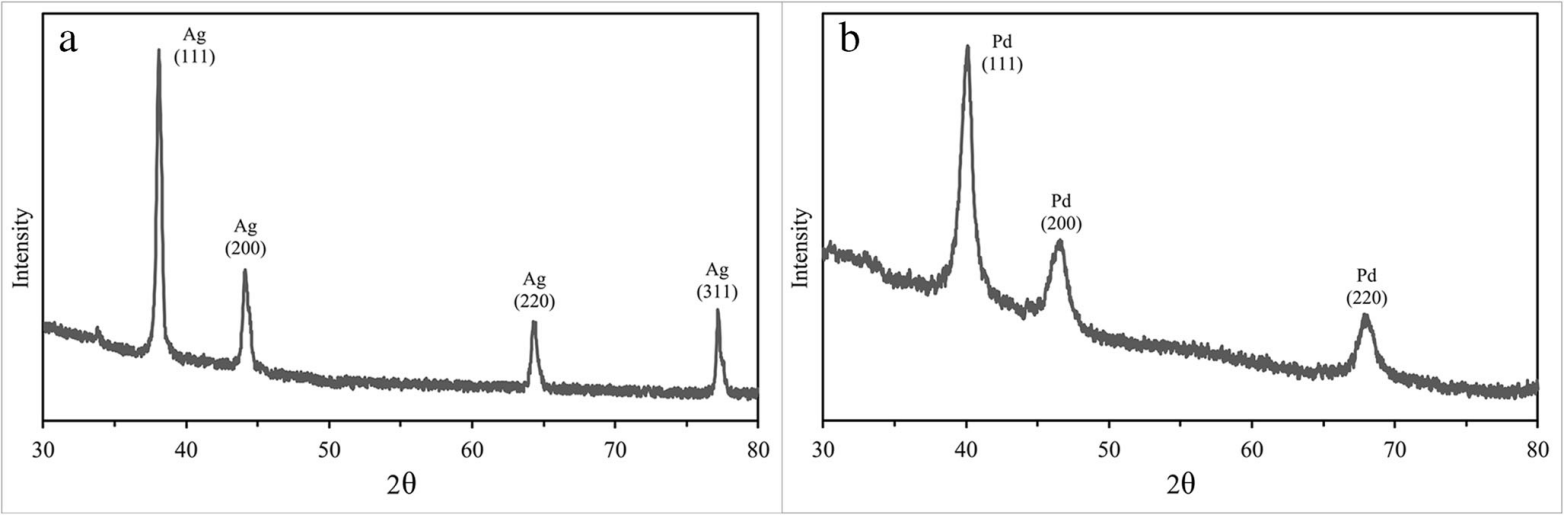
XRD patterns typical for Ag**°**NPs obtained with *M. quaylei* MT^T^ and *M. quaylei* SM^R^ (**a**) and for Pd**°**NPs obtained with *M. quaylei* SM^R^ (**b**)

Both cultures of methylotrophic bacteria can generate sufficiently large silver nanoparticles after the addition of 0.1 mM Ag(NH_3_)_2_NO_3_ solution for several minutes. Analysis of the size distribution showed that in the presence of wild-type cells, Ag°NPs have a size ranging from 16 to 100 nm with a bimodal distribution (with the primary fraction 45 nm). With the culture *M. quaylei* SM^R^, nanoparticles are somewhat larger, predominantly ranging from 25 to 120 nm with a bimodal distribution and the primary fraction of approximately 70 nm (Figs. 2 and 3, Table 1). It is significant that most of the silver NPs are adsorbed on the cells or somehow associated with them. It can also be noted that the reduced secretion of exopolysaccharides and the extremely low hydrophobicity of the cell surface in the culture of *M. quaylei* SM^R^ promote the formation of both silver and palladium nanoparticles (Table 1). The XRD patterns are shown in Fig. 7 and also confirmed the formation of Ag^0^ and Pd^0^NPs with face centered cubic (fcc) crystalline structure.

In general, this study shows for the first time the ability of obligate methylotrophic bacteria of the species *M. quaylei* to form noble metal nanoparticles; unlike the culture of the wild type (*M. quaylei* MT^T^), the culture of the isogenic streptomycin-resistant mutant (*M. quaylei* SM^R^) reduces and forms nanoparticles from both silver and palladium cations. Such a clear difference in the formation of biogenic silver and palladium nanoparticles (noble metals of differing cationic valences) indicates that the DBNG method allows researchers using several metals under similar conditions to distinguish very closely related cultures. This method would be an analog of a “variegated series” in the comparison of microbial species or cultures. It can be postulated that the assessment of the interaction of microbial cells with the salts of several metals may be useful for more complete characterization of closely related drug-resistant cultures of clinical isolates.

It has been shown that both cultures of the obligate methylotrophic bacteria *M. quaylei* (wild-type MT^T^ culture and streptomycin-resistant mutant SM^R^) can reduce silver salts and form Ag°NPs directly in a minimal synthetic medium with methanol as the only organic carbon source. In contrast to the wild-type culture, the isogenic streptomycin-resistant mutant *M. quaylei* SM^R^ can generate nanoparticles from reduced palladium atoms.

It can be expected that the assessment of the interaction of the microbial cells with various metal salts may be useful for more complete characterization of closely related drug-resistant cultures of clinical isolates.

## Abbreviations

NPs: Nanoparticles
Ag**°**NPs: Silver nanoparticles
Pd**°**NPs: Palladium nanoparticles
DBNG: Detection of biogenic nanoparticles generation
PQQ: Pyrroloquinoline quinone
EDTA: Ethylenediaminetetraacetic acid
CFU: Colony forming unit
EPS: Exopolysaccharides
